# From copper homeostasis to cuproptosis: a new perspective on CNS immune regulation and neurodegenerative diseases

**DOI:** 10.3389/fneur.2025.1581045

**Published:** 2025-05-29

**Authors:** Luhao Li, Liangzhen Lv, Zhaodi Wang, Xianbao Liu, Qingyi Wang, Hui Zhu, Bei Jiang, Yapeng Han, Xue Pan, Xueming Zhou, Li Ren, Zhuo Chang

**Affiliations:** ^1^Second Clinical Medical School, Heilongjiang University of Chinese Medicine, Harbin, China; ^2^Graduate School, Heilongjiang University of Chinese Medicine, Harbin, China; ^3^Yichun Central Hospital, Yichun, Heilongjiang, China; ^4^Yueyang Hospital of Integrated Traditional Chinese and Western Medicine, Shanghai University of Traditional Chinese Medicine, Shanghai, China; ^5^School of Basic Medical Sciences, Heilongjiang University of Chinese Medicine, Harbin, China; ^6^Third Affiliated Hospital, Beijing University of Chinese Medicine, Beijing, China; ^7^Shunde Women and Children’s Hospital, Guangdong Medical University, Foshan, China

**Keywords:** copper homeostasis, cuproptosis, neurodegenerative diseases, immune response, cell death mechanisms

## Abstract

Copper, an essential trace element for the human body, plays a key role in energy metabolism, mitochondrial respiration, redox reactions, and neural signal transmission. The recently proposed concept of “cuproptosis” has further revealed the unique status of copper in cellular regulation: when copper abnormally accumulates within cells, it can directly bind to the lipoylated proteins of the mitochondrial TCA cycle, triggering protein aggregation and metabolic disorders, ultimately leading to cell death. This form of cell death plays an important role in various neurodegenerative diseases of the central nervous system, such as Alzheimer’s disease (AD), Parkinson’s disease (PD), amyotrophic lateral sclerosis (ALS), Huntington’s disease (HD), and stroke. This review summarizes recent research on the mechanisms of cuproptosis, providing new perspectives and a theoretical basis for understanding the pathogenesis of these neurodegenerative diseases.

## Introduction

Copper, an essential trace element, is primarily absorbed through dietary intake and enters intestinal epithelial cells (1). From there, it is distributed and transported throughout the body by various transport proteins (e.g., Ctr1 and Ctr2) and copper chaperone proteins (e.g., ATOX1, CCS, and COX17) (2). The liver, as the primary storage site for copper, regulates its distribution to tissues or excretion via bile (3). Copper is essential in physiological processes such as mitochondrial respiration, cellular energy metabolism, electron transport, and neurotransmitter synthesis (4). Copper levels are closely linked to immune function and typically remain stable within a certain range under normal physiological conditions, primarily in the brain, liver, and bones (5, 6). However, both excess and deficiency of copper can lead to pathological changes, adversely affecting health (7).

Cuproptosis is a newly identified form of cell death first reported and named by Tsvetkov et al. (8). The study found that copper ions bind directly to the lipoylated proteins of the mitochondrial TCA cycle, causing protein aggregation and metabolic disruptions that ultimately result in cell death (8). Among these key proteins, lipoylated proteins (particularly the PDH complex) and Fe–S cluster proteins serve as copper-binding sites and play critical roles in the cell (9). When copper ion levels rise, the functions of these proteins are compromised, accompanied by protein toxicity stress (such as HSP70 induction) and the collapse of mitochondrial metabolism, ultimately leading to cell death (7). FDX1, identified as a central regulatory gene of cuproptosis, promotes copper-dependent cell death by regulating the protein lipoylation process (10). This copper ion overload-induced programmatic cell death mechanism differs from all known forms of programmed cell death (such as apoptosis, ferroptosis, pyroptosis, and necroptosis) (9). The key events involve abnormal copper accumulation in cells and mitochondria, leading to impaired mitochondrial function, enhanced oxidative stress, and metabolic dysregulation (11).

Neurodegenerative diseases (NDD) are caused by degeneration or demyelination of neurons in the brain or spinal cord and can result from various etiologies, such as abnormal protein aggregation, misfolded protein propagation, genetic mutations, and epigenetic modifications (12). Examples of NDDs include Alzheimer’s disease (AD), Parkinson’s disease (PD), Huntington’s disease (HD), amyotrophic lateral sclerosis (ALS), spinal muscular atrophy (SMA), multiple sclerosis (MS), traumatic brain injury (TBI), and stroke. Studies have shown that between 1996 and 2016, the number of people with Parkinson’s disease (PD) doubled (13). Currently, approximately 47 million people worldwide live with dementia, with projections suggesting this number could triple to around 131 million by 2050 (14). The precise pathogenesis of these diseases remains incompletely understood (14).

This article reviews the role of cuproptosis in central nervous system immune responses and its potential mechanisms in neurodegenerative diseases. By investigating these mechanisms, this review provides new insights into disease pathogenesis and suggests potential directions for prevention and treatment through the lens of cuproptosis.

### Copper absorption

Copper in the human body is primarily obtained from organ meats and shellfish, with adults recommended to consume between 0.8 to 2.4 mg of copper daily to maintain balance (15). Copper is primarily absorbed through the duodenum, where it enters intestinal epithelial cells via copper transporter 1 (Ctr1) and divalent metal transporter DMT1 (DMT1) (2). Once inside these cells, copper is either distributed to copper chaperone proteins or stored as metallothionein (MT) and glutathione (GSH) (16). An animal study demonstrated that in mice lacking the copper transporter Ctr1, although copper accumulated significantly in intestinal epithelial cells, it could not be converted into a bioavailable form and thus failed to perform its normal physiological functions (17). These Ctr1-deficient mice exhibited marked growth retardation and reduced survival rates early after birth, likely due to insufficient copper absorption and subsequent impairment of copper-dependent enzymes and proteins (18). Partial correction of growth and survival defects was achieved by copper supplementation, underscoring the vital role of copper in early development (17).

Research indicates that when copper levels are insufficient, Ctr1 expression increases in key tissues (e.g., intestine, kidney, and brain), thereby promoting copper uptake and recycling to meet the body’s copper needs (19). Conversely, when copper levels are excessive, Ctr1 expression decreases, limiting excessive copper absorption and preventing copper toxicity (18). Such regulation allows cells and the organism to maintain copper homeostasis under varying copper supply conditions (20). Another study reached a similar conclusion, demonstrating that copper homeostasis is maintained by regulating the expression of Sp1 (Specificity protein1) and hCtr1 (21). In copper excess, hCtr1 is upregulated to enhance copper uptake while Sp1 expression is inhibited; when copper is deficient, Sp1 is upregulated to increase hCtr1 expression and thus boost copper intake (22). By means of a feedback mechanism, Sp1 and hCtr1 regulate each other, maintaining a balanced intracellular copper level (21).

### Copper storage and transportation

Copper absorbed by intestinal epithelial cells via the transporter Ctr1 is guided by the copper chaperone Atox1 to ATPase copper transporter 7A (ATP7A) (23). ATP7A, located on the basolateral side of the epithelial cells, actively transports copper into the bloodstream (16). In the bloodstream, copper ions bind to proteins rather than circulating as free ions (23). Human serum albumin (HSA) and ceruloplasmin (CP) maintain the dynamic equilibrium of copper in the serum via distinct binding properties: CP stabilizes and stores most of the copper, while HSA is the primary carrier for exchangeable copper, aiding in copper transport and distribution (24). Specifically, about 75% of copper ions are bound to ceruloplasmin in a non-exchangeable form, whereas approximately 25% are bound to HSA in an exchangeable form (24).

Subsequently, the majority of copper ions travel via the portal vein to the liver, the central organ for copper metabolism by regulating absorption and biliary excretion (25). Metallothionein (MT), a molecule rich in thiol groups and with high affinity for copper ions, helps maintain copper homeostasis by storing and regulating copper ions (26). When cells require copper, MT releases these ions (26). This mechanism allows MT to prevent oxidative stress caused by copper overload while preserving physiological copper balance (27). ATP7B supports copper homeostasis, especially in the kidney, by facilitating copper efflux and modulating intracellular copper levels, redistributing copper from the liver to the bloodstream as needed (28).

### Copper elimination

ATP7A and ATP7B are P1B-type ATPases responsible for the intracellular distribution, excretion, and storage of copper (29). When copper levels rise, these transporters relocate from the trans-Golgi network (TGN) to the cell membrane or lysosomes, promoting copper excretion or storage; when copper levels fall, they return to the TGN to restore the supply of copper for enzyme activity (25). Under normal physiological conditions, copper is primarily transported into bile by ATP7B and excreted with bile, with only about 2% excreted through urine (30).

**Figure 1:**
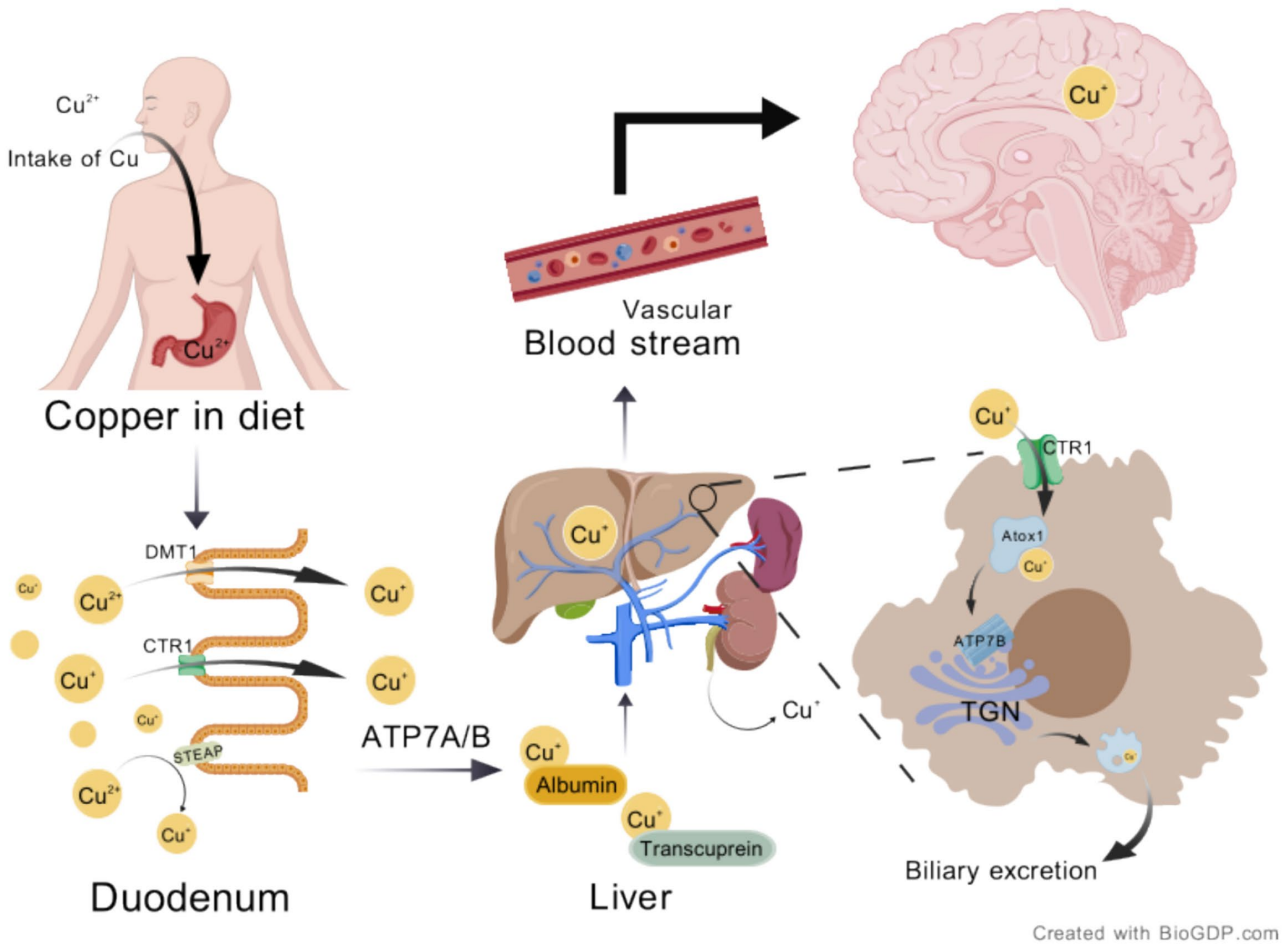
Schematic of normal copper metabolism. Copper from food is first reduced from Cu^2+^ to Cu^+^ by STEAP, then absorbed by enterocytes through the transporter Ctr1. In certain cases, DMT1 also participates in copper transport and absorption. Subsequently, copper is transported to the portal vein via the ATP7A transporter, where it binds to plasma proteins such as albumin and ceruloplasmin for further transport to the liver. After processing or transporting copper, the liver releases it into the bloodstream, which then enters the brain circulation, while excess copper is excreted through bile. Created with BioGDP.com (236). ATP7A, Cu-transporting ATPase 1; ATP7B, ATPase Cu-transporting beta; Ctr1, Cu transporter 1; Cu, copper; DMT1, divalent metal transporter 1; STEAP, six segment transmembrane epithelial antigen of prostate; Atox1, antioxidant 1; TGN, trans-Golgi network.

An animal experiment demonstrated that deficiency of MURR1 led to copper accumulation in the liver and reduced copper excretion into bile, ultimately causing cirrhosis (31). This result suggests that MURR1 may participate in extracellular copper excretion by regulating ATP7B-mediated copper discharge (32). In Wilson’s disease (WD), mutations in the ATP7B gene impair its function, hindering copper excretion (33). Certain mutations cause ATP7B to accumulate in the endoplasmic reticulum, impairing its proper localization to the Golgi apparatus or cell membrane, preventing copper from being excreted in bile and leading to its buildup in the liver, brain, and other tissues (29). This accumulation leads to copper toxicity symptoms characteristic of Wilson’s disease (34) (Figure 1).

### Systemic copper homeostasis

Copper is an essential trace nutrient in the human body, forming an integral part of various key metabolic enzymes like copper-zinc superoxide dismutase (SOD1), cytochrome c oxidase (CCO), and lysyl oxidase (LOX) (35). Copper is widely distributed in the body, primarily in the skeleton, liver, and kidneys (19). The skeleton contains the highest copper concentration, about 46 mg, while the kidneys contain relatively less, around 3 mg (36). Because copper exists in two oxidation states (Cu^+^ and Cu^2+^) and plays a critical cofactor role in the body’s redox systems (37). Excessively high or low copper levels can result in cytotoxicity and pathological changes (30). Maintaining copper levels within an appropriate range is essential (37).

### Intracellular copper homeostasis

Copper uptake, distribution, and excretion within cells is a dynamic equilibrium regulated by metabolic demands, differentiation status, and environmental factors (38). Copper chaperone proteins like Atox1 and CCS transfer copper from Ctr1 to ATP7A/ATP7B or to copper-dependent enzymes, ensuring the efficient allocation of copper (39). An animal study showed that copper, via Atox1 as a transcription factor within the cell nucleus, binds to the Cyclin D1 promoter and promotes Cyclin D1 expression, thereby driving cell-cycle progression and cell proliferation (40). These findings indicate that Atox1’s nuclear translocation and structural domain integrity are essential for these processes (41).

CCS forms a heterodimer with superoxide dismutase 1 (SOD1), facilitating copper ion transfer from CCS to SOD1 and activating its superoxide dismutase function (42). SOD1 is a key antioxidant enzyme widely present in eukaryotic cells (43). It catalyzes the conversion of superoxide radicals (O_2_^−^) into hydrogen peroxide (H_2_O_2_) and oxygen (O_2_), a reaction critical for maintaining redox balance in cells (44). By doing so, it prevents oxidative stress and cellular damage caused by the accumulation of superoxide radicals (44). A recent study has shown that in SOD1-knockout mice, the absence of CuZn superoxide dismutase (CuZnSOD) led to excessive superoxide accumulation, causing oxidative stress in mitochondria and the cytoplasm (45). This oxidative stress impaired multiple antioxidant enzymes (e.g., MnSOD and GPx) and led to widespread oxidative damage in the liver, including protein oxidation, lipid peroxidation, and DNA damage (46). Significant pathological changes, including the formation of hepatocellular carcinoma (HCC), occurred in hepatocytes (47).

The protein encoded by Cox17 is crucial for copper ion transport, delivering copper ions from the cytoplasm to mitochondria, aiding in cytochrome c oxidase (CCO) assembly and activity (48). The mitochondrial inner-membrane protein PiC2 (SLC25A3) can bind copper ions and acts as a mitochondrial copper transporter, delivering copper into the organelle and promoting the metallation of cytochrome c oxidase (CCO) in eukaryotic cells (49). Although there is not yet sufficient evidence to definitively classify COA6 as a copper chaperone, studies indicate that COA6 can indirectly facilitate copper binding by promoting the reduction of disulfide bonds in the copper-coordinating cysteine residues of COX2 and SCO1 (49). Another study reported that Cox17 specifically transfers copper ions to Sco1 and Cox11 during the assembly of cytochrome c oxidase (CCO), functioning as a key copper donor (50). This mechanism ensures proper metallation of CCO and the maintenance of its normal biological function (51). An animal experiment revealed that loss of COX17p function leads not only to the failure of the mitochondrial respiratory chain but also to severe developmental defects in early embryogenesis (52). COX17p deficiency does not cause immediate lethality in early embryonic development, but as development progresses, impaired copper ion transport into mitochondria disrupts cellular energy supply, ultimately proving fatal (53).

**Figure 2:**
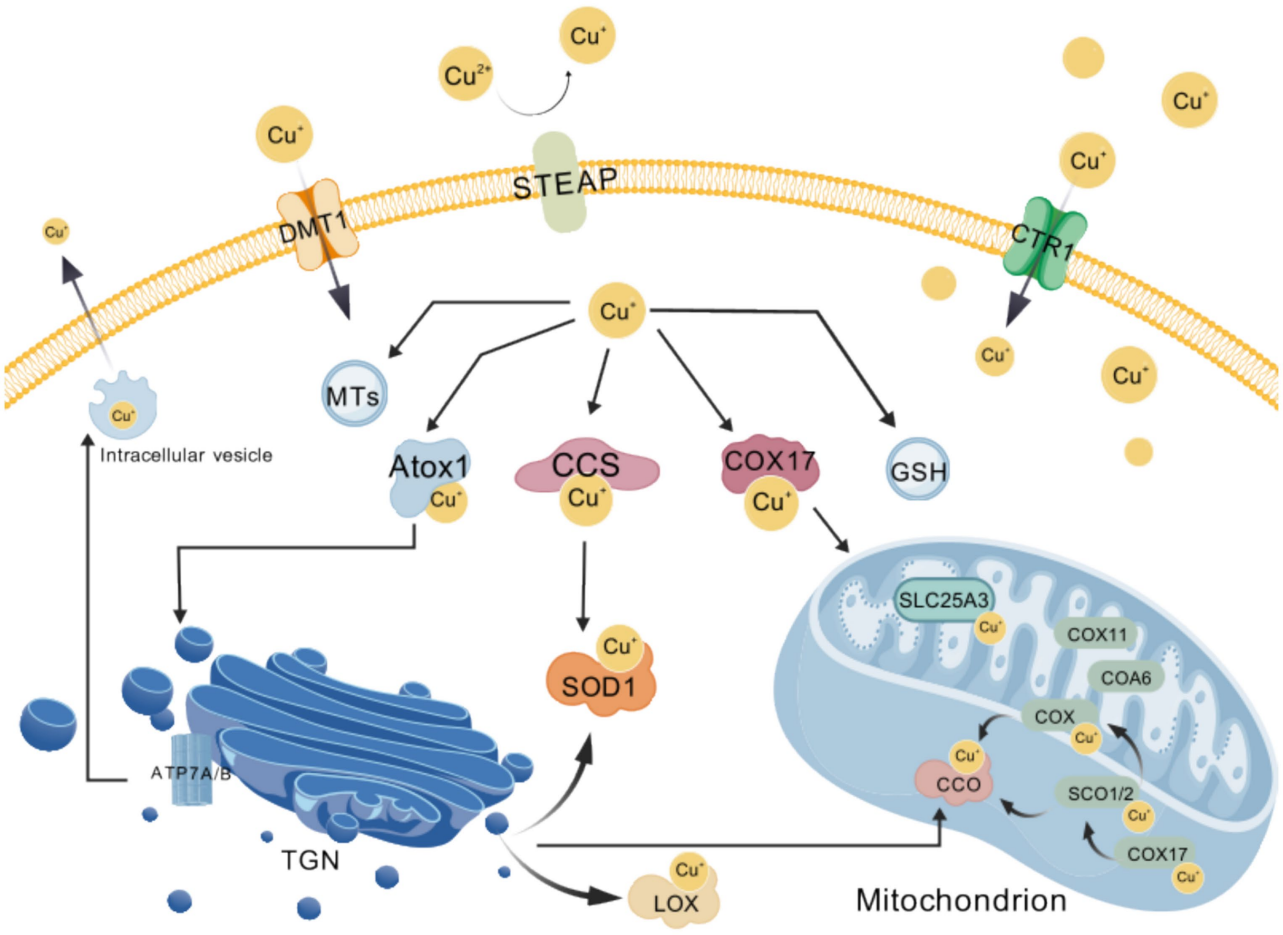
Overview of cellular copper homeostasis. Exogenous cupric ions (Cu^2+^) are first reduced to cuprous ions (Cu^+^) by the STEAP family of metalloreductases and subsequently imported into the cytosol via the membrane transporters CTR1 and DMT1; the resulting Cu^+^ is then precisely delivered to distinct targets by the copper chaperones ATOX1, CCS and COX17, while excess free copper is transiently sequestered by metallothioneins (MTs) and glutathione (GSH) to buffer cellular levels; when copper must be expelled, the P-type ATPases ATP7A and ATP7B pump Cu^+^ either out of the cell or into secretory vesicles; within the mitochondrial intermembrane space, SCO2 and COA6 cooperatively adjust the redox state of SCO1 so that it can bind Cu^+^ and hand the metal to cytochrome-c oxidase (CCO), whereas the inner-membrane carrier SLC25A3 transports Cu^+^ into the matrix to support this metallation process; these complementary mechanisms of uptake, storage, trafficking and efflux collectively ensure that copper-dependent enzymes—including CCO, lysyl oxidase (LOX) and superoxide dismutase 1 (SOD1)—acquire their essential metal cofactor, thereby sustaining cellular energy metabolism and antioxidant homeostasis. Created with BioGDP.com (236). CCS, copper chaperone for superoxide dismutase; COX17, cytochrome-c oxidase copper chaperone 17; MTs, metallothioneins; GSH, glutathione; SCO2, synthesis of cytochrome-c oxidase 2; COA6, cytochrome-c oxidase assembly factor 6; SCO1, synthesis of cytochrome-c oxidase 1; CCO, cytochrome-c oxidase, complex IV; SLC25A3, solute carrier family 25 member 3, mitochondrial phosphate carrier; LOX, lysyl oxidase; SOD1, superoxide dismutase 1.

Copper-regulated kinases (e.g., ULK1, ULK2, and MEK1) and the cAMP-degrading enzyme PDE3B mediate copper-dependent signaling that influences autophagosome formation, cell proliferation, and metabolism (54). Cytoplasmic glutathione affects both the rate of copper uptake and the oxidation state of Atox1, thereby modulating copper distribution (25). SLC31A1 (Ctr1) is the principal high-affinity copper transporter responsible for most cellular copper uptake (19). When intracellular copper levels rise, Ctr1 is internalized from the plasma membrane into endosomes through clathrin- and dynein-dependent endocytosis, thus reducing copper uptake; when copper levels fall, Ctr1 is redirected back to the plasma membrane via the retromer complex, reinstating its copper-uptake function (25) (Figure 2).

### Copper homeostasis and cuproptosis in neurodegenerative diseases of the central nervous system

#### Alzheimer’s disease

Alzheimer’s disease (AD) is a neurodegenerative disorder of the central nervous system, mainly characterized by progressively worsening cognitive impairment and behavioral abnormalities (55). It is the most common form of dementia (56). Studies predict that by 2050, AD will affect 115 million people worldwide (57).

While the exact cause of Alzheimer’s disease remains unclear, multiple hypotheses have been proposed to explain its pathogenesis (55). An ATN (amyloid-β, tau protein, and neurodegeneration) framework based on biomarkers, emphasize the presence of β-amyloid (Aβ) and phosphorylated tau protein to confirm AD (58). According to the “amyloid cascade” hypothesis, the initial event in AD is the misfolding and aggregation of Aβ, which triggers a series of pathological changes, including abnormal tau phosphorylation, neuroinflammation, vascular abnormalities, and neurodegeneration (59). Accumulation of Aβ within neuronal mitochondria increases ROS levels and disrupts the mitochondrial membrane potential, resulting in energy metabolic disorders that lead to apoptosis or necroptosis, severely compromising neuronal function and survival (60).

Other studies suggest that oxidative damage—through excessive reactive oxygen species (ROS)—leads to metabolic disturbances in neurons and is closely associated with Aβ accumulation, creating a vicious cycle (61). Additionally, Aβ promotes the abnormal phosphorylation of tau protein, causing microtubule disassembly and neuronal dysfunction (62, 63). Hyperphosphorylated tau is strongly associated with neurofibrillary tangle (NFT) formation, with tau phosphorylation directly affecting its binding to microtubules (64, 65). Hyperphosphorylated tau loses its microtubule-stabilizing function and forms NFTs, further exacerbating neurodegenerative changes (66, 67). Moreover, Aβ can activate signaling pathways such as p38MAPK to induce abnormal tau phosphorylation; once Aβ deposition reaches a certain level, it accelerates tau pathology and promotes the formation of amyloid plaques, ultimately causing a gradual decline in cognitive function (68, 69). In addition, theories such as the neuroinflammation hypothesis and the cholinergic hypothesis have been proposed to further explain the pathogenesis of AD (70).

Dysregulated copper metabolism is closely linked to AD progression (71). Studies show that in the brains of AD patients, copper distribution is abnormal; both copper excess and copper deficiency have been observed in various regions of the brain (72). Excess copper primarily exacerbates cognitive impairment by increasing the expression of amyloid precursor protein (APP) and β-site APP cleaving enzyme 1 (BACE1), promoting the accumulation of amyloid β (Aβ) (73). In particular, copper exposure significantly increased the mRNA and protein expression of APP and BACE1 in the hippocampus, leading to the accumulation of Aβ42, which further inhibited hippocampal long-term potentiation (LTP), a mechanism crucial for learning and memory (74). Copper exposure also disrupted synaptic plasticity by generating oxidative stress, leading to memory loss (74). In the cortex, although copper exposure did not significantly increase the expression of ADAM10, copper negatively affected the Aβ clearance mechanism (75). Copper may influence the expression of Aβ degrading enzymes such as neprilysin (NEP) and insulin-degrading enzyme (IDE), leading to insufficient Aβ clearance, which in turn affects decision-making, planning, and executive functions (76). Studies also suggest that reduced copper levels in the hippocampus may impair synaptic plasticity and neuronal growth, leading to memory decline (77). A randomized controlled trial found that copper concentration in the frontal cortex was significantly lower in Alzheimer’s disease (AD) patients compared to healthy controls, with this change mainly occurring in soluble components, leading to impaired cognitive control, such as difficulties in decision-making and attention deficits (78). This indicates a localized reduction in copper in the brains of AD patients, affecting only soluble components, while other regions such as peripheral membranes, vesicular material, and membrane components showed no significant differences (79). In the frontal cortex, copper overload induces oxidative stress and cell damage by increasing free copper, which impacts cognitive abilities and memory function (80). Although the antioxidant HT can restore copper levels and improve behavioral performance, the increase in calcium ion levels may limit these improvements, suggesting that copper metabolic imbalance affects cognitive function through a series of biochemical reactions (80). Excess copper in the medial temporal cortical region exacerbates oxidative stress, promoting the aggregation and deposition of β-amyloid, thereby worsening cognitive decline (55). An animal study showed that copper concentrations in the brainstem of AD model mice were significantly increased, particularly in transgenic AD mice, where copper levels were significantly higher in both young and old mice compared to healthy controls (Mann–Whitney *U*, *p* = 0.016), indicating that the brainstem may be a key area for early pathological changes in AD (81). Copper concentrations in the cerebellum also increased with age, but in AD mice, while copper levels did not significantly change (Mann–Whitney *U*, *p* = 0.063), significant changes in copper isotope ratios were observed (Mann–Whitney *U*, *p* = 0.016), with a notable enrichment in copper isotope ratios in AD transgenic mice (Δ65Cu = 0.33‰) (81). This suggests that copper metabolism imbalance may alter the redox status of the cerebellum, thereby affecting its function (81). In the TgCRND8 mouse model, β-amyloid plaques predominantly accumulate in various regions of the cerebral cortex, where copper concentrations were significantly higher, with copper binding to Aβ peptides to promote Aβ aggregation and neurotoxicity (82). However, in areas surrounding the plaques, copper concentrations were lower, which may be related to the upregulation of ATP7A copper transport protein in activated microglia, particularly in the regions around the plaques (78). Activated microglia, through the secretion of inflammatory cytokines like interferon-γ (IFN-γ), participate in inflammatory responses, affecting copper uptake and transport. After IFN-γ stimulation, ATP7A proteins are redistributed to intracellular vesicles, suggesting copper accumulation inside the cell. Microglia may “sequester” copper to reduce the negative interaction between copper and Aβ, thereby exerting a protective effect (82). In AD patients, the concentration of free copper (i.e., copper not bound to ceruloplasmin) is elevated, and free copper can cross the blood-brain barrier (BBB) to enter the cerebrospinal fluid (CSF) and cerebral cortex, where it interacts with Aβ to promote its aggregation (83). This copper metabolic dysfunction is closely associated with clinical symptoms, neuronal damage, and changes in biomarkers in the CSF (83). The increase in free copper is also closely linked to neuroinflammation, as it promotes the activation of microglia and astrocytes, triggering an inflammatory response in the brain, which further exacerbates neuronal damage and cognitive decline (83). Studies suggest that copper deficiency in the basal forebrain and thalamus may impair cholinergic system function, negatively affecting behavior and cognition (84). Furthermore, abnormalities in copper levels in the anterior cingulate cortex, a key brain area involved in emotional regulation and cognitive conflict, may also be closely related to cognitive symptoms and emotional changes in AD (85). Although the cerebellum is less affected in AD pathology, abnormal copper levels in this region may be associated with impaired motor coordination and cerebellar function in some AD patients (86). Other studies have found that abnormal copper distribution in the cerebellum, by affecting ion transport activity, membrane potential changes, and intracellular calcium levels, may directly influence the electrophysiological activity of neurons, leading to cognitive and behavioral changes (87). This phenomenon of copper imbalance is part of the AD pathology, referred to as the “CuAD hypothesis,” which suggests that both copper excess and deficiency lead to neuronal damage and functional impairment (88). Moreover, abnormal copper accumulation in the basal ganglia may disrupt the normal function of neurotransmitter systems, particularly those related to dopamine (89). Dopaminergic neurons in the basal ganglia play an important role in motor control, reward mechanisms, and emotional regulation (90). Research has shown that copper overload may alter dopamine synthesis, release, and clearance processes through interaction with dopamine receptors or by affecting dopamine transporter function, leading to dopamine metabolism abnormalities and impacting basal ganglia function (91). Insufficient or excessive activation of dopamine can directly lead to motor dysfunctions such as tremors and bradykinesia, which are common motor abnormalities observed in AD patients (92). Copper overload in the basal ganglia not only affects motor functions but may also further interfere with emotional and motivational regulation (93). Another important function of the basal ganglia is regulating emotions and decision-making (94). Copper overload may influence this function in two ways: first, the direct toxicity of copper to neurons may damage basal ganglia circuits, particularly those related to emotion and motivation; second, copper may alter the balance of neurotransmitter systems, leading to emotional regulation disorders, further contributing to depression, anxiety, and other emotional issues, which are very common in AD patients (95). AD patients often exhibit emotional changes, which are closely related to basal ganglia dysfunction (94).

These specific manifestations of copper abnormalities in different pathological stages and brain regions of AD suggest that copper’s role in AD is not merely confined to simple concentration changes within brain regions but rather influences neuronal function and the stability of neural circuits through complex biochemical mechanisms, thereby playing a critical role in the cognitive impairment and behavioral changes observed in AD (96).

Cu^2+^ coordinates with the histidine (His) and tyrosine (Tyr) residues of Aβ, affecting its aggregation pathway and forming two main aggregate types: amyloid-like fibrils and Cu^2+^-induced aggregates (97, 98). Cu^2+^-induced aggregates generate dityrosine, triggering oxidative stress and increasing neurotoxicity (99). When the Cu^2+^:Aβ molar ratio is below 1:1, Cu^2+^ primarily promotes the formation of amyloid-like fibrils; at higher molar ratios, it favors Cu^2+^-induced aggregates, which hinder fibrillization (100). Cu^2+^ binding also promotes the conversion of tau from its native structure into a conformation that is more prone to aggregation, providing a basis for pathological tau aggregations such as neurofibrillary tangles (NFTs) (30). Furthermore, Cu^2+^ induces nitrotyrosination of lipoprotein receptor-related protein 1 (LRP1), marking it for proteasomal degradation and thereby reducing LRP1 levels, which in turn lowers Aβ clearance efficiency (101).

A meta-analysis revealed significant alterations in trace elements in AD patients, notably copper (serum), iron (plasma), zinc (hair), and selenium (plasma), with standardized mean differences (SMD) of 0.37, −0.68, −0.35, and −0.61, respectively (102). Another meta-analysis showed that copper content in the brain tissue of AD patients decreases, while copper and non-ceruloplasmin bound copper (non-Cp Cu) levels in serum/plasma increase (103). Moreover, the AG haplotype of the ATP7B gene is associated with increased susceptibility to AD, indicating that AD patients may fail to maintain proper copper metabolism and that certain individuals carrying the ATP7B AG haplotype may be more prone to copper imbalance (103). This suggests that copper dyshomeostasis could represent a disease subtype in AD.

Research indicates that iron, copper, and zinc each bind to β-amyloid (Aβ) to modulate its aggregation and toxicity, inducing oxidative stress and programmed cell death (including ferroptosis and cuproptosis) and further aggravating neuronal damage (70, 104). At the same time, iron and copper contribute to neurofibrillary tangle (NFT) formation by activating tau-protein kinases (e.g., GSK3β) and facilitating excessive tau phosphorylation (70). One study revealed that copper ions bind to the histidine residues of Aβ, forming a non-β-folded Aβ/Cu complex that inhibits fibrillization and blocks the formation of typical amyloid fibrils (70). This process may, in early stages of AD, influence the aggregation patterns of Aβ and the development of senile plaques, thereby driving disease pathology (105).

The role of copper in Alzheimer’s disease (AD) extends beyond the accumulation of Aβ and tau; copper also plays a significant role in disease progression by regulating immune system functions (58). Copper’s role in AD is first manifested in its regulation of immune molecules (59). Studies show that molecules like Aβ, tau, and ApoE maintain neural homeostasis under normal conditions, but in AD, their functions change, and they shift to exert antimicrobial effects (106). For example, Aβ, as a natural antimicrobial peptide (AMP), forms fibrous structures through aggregation, interacting with pathogens to defend against infections (106). In this process, copper binds to Aβ, promoting its aggregation and enhancing Aβ’s antimicrobial activity (106). However, prolonged copper accumulation can lead to excessive aggregation of Aβ, which in turn triggers neurotoxicity, causing immune system dysfunction and exacerbating neurodegenerative changes (106).

Additionally, copper significantly affects the activity of immune cells (107). In the brains of AD patients, abnormal distribution of copper leads to overactivation of microglial cells and astrocytes (107). The abnormal response of these immune cells to pathogen invasion aggravates neuroinflammation (107). The loss of function of copper-dependent enzymes, such as cytochrome c oxidase and ceruloplasmin, in AD further disrupts iron homeostasis and redox reactions, thereby intensifying neuroinflammatory responses (107).

Copper’s role in immune regulation is also reflected in its control over immune cell function (59). In AD, copper binds to Aβ to form a more stable redox complex, which not only enhances Aβ’s antimicrobial activity but also leads to copper depletion, further affecting the normal function of the nervous system (101). Copper deficiency reduces the activity of copper enzymes such as ceruloplasmin, which are closely related to neuroimmune functions and iron homeostasis, thereby exacerbating neurodegenerative changes (106).

Copper interacts with key proteins in the immune system to regulate immune responses (103). Studies have shown that copper promotes the release of interferon-γ (IFN-γ), increases copper uptake in microglial cells, and enhances ATP7A expression, which helps limit Aβ aggregation and improves its clearance (103). At the same time, copper regulates the M1/M2 shift in microglial cells, exerting its dual effect (107). In some cases, copper suppresses the generation of nitric oxide (NO), promoting the transformation of microglial cells from the pro-inflammatory M1 type to the neuroprotective M2 type, thus alleviating neuroinflammation and promoting neuroprotection (107). This indicates that the immune regulatory role of copper depends on its physiological concentration and distribution, with appropriate copper levels helping to suppress excessive immune responses and exert protective effects (107).

In summary, copper’s immune regulatory role in AD is dual in nature, potentially promoting harmful inflammatory responses but also, under appropriate conditions, exerting anti-inflammatory and neuroprotective effects (107). The dysregulation of copper homeostasis leads to immune dysfunction by affecting the function of immune molecules, the activation state of immune cells, and the deficiency of copper enzymes, which in turn drives the progression of neurodegenerative changes (59). Both copper overload and deficiency can affect the activity of microglial cells, astrocytes, and other immune cells, intensifying neuroinflammation and ultimately influencing the course of AD (106). Therefore, regulating copper homeostasis is a critical component of the pathological mechanism of AD, offering new directions and potential targets for AD treatment (59).

#### Amyotrophic lateral sclerosis

Amyotrophic lateral sclerosis (ALS) is a neurodegenerative disease marked by the degeneration of motor neurons (108). Common symptoms include skeletal muscle weakness, atrophy, fasciculations, and bulbar palsy (109). Progressive paralysis eventually leads to respiratory failure, with death typically occurring within about 3 years of onset (110). The exact etiology of ALS remains unclear, potentially involving genetic factors, immune system dysfunction, lifestyle choices, and metabolic imbalances (111). Despite extensive studies on its pathogenesis, challenges in ALS diagnosis and treatment persist, with therapeutic outcomes still suboptimal (108). In recent years, an increasing number of studies have underscored the importance of targeted therapy for maintaining copper homeostasis (112, 113).

Research has shown that misfolded mutant SOD1 can bind to proteins on the mitochondrial outer membrane (OMM), such as Bcl-2 and VDAC1, thereby causing mitochondrial dysfunction, increased oxidative stress, and activation of apoptotic pathways (114, 115). These changes promote cell apoptosis and ultimately lead to the death of motor neurons, contributing to the onset of ALS (116). Meanwhile, copper—regulating SOD1 activity—has gained recognition for its role in the aggregation and abnormal function of SOD1 (115). Copper metabolism imbalances may exacerbate SOD1 misfolding, forming insoluble aggregates and accelerating ALS pathology (117).

An animal study demonstrated that tetrathiomolybdate (TTM) provides protective effects in ALS by modulating superoxide dismutase 1 (SOD1) and copper metabolism, restoring abnormally elevated copper levels in the spinal cord (118). TTM significantly reduced insoluble SOD1 aggregates and suppressed SOD1 enzyme activity, thereby slowing the pathological progression of ALS (119). A randomized controlled trial showed significantly lower copper levels in the cerebrospinal fluid (CSF) of ALS patients compared to healthy controls, particularly in spinal-onset ALS (109). Research reports that certain SOD1 mutations (e.g., A4V) reduce enzymatic activity and copper-binding affinity (120). Under zinc-deficient conditions, mutant SOD1 can generate highly active hydroxyl radicals and peroxynitrite via abnormal copper chemistry, further exacerbating neuronal oxidative damage and protein nitration (120). This same study indicated that dysregulated copper metabolism promotes SOD1 aggregation and disulfide bond instability, further aggravating ALS pathology (121).

Copper chaperone for SOD1 (CCS) regulates copper uptake and structural maturation of SOD1 (122). One study found a positive correlation between CCS expression and survival in ALS patients, suggesting that higher CCS expression may prolong survival and confer protective effects in ALS pathogenesis (123). A randomized controlled study revealed higher expression of copper metabolism-related genes (CuRGs) in ALS patients, highlighting their pivotal role in ALS onset (111). Moreover, by analyzing differing CuRG expression profiles, the study classified ALS patients into two distinct pathological subtypes with marked differences in immune characteristics and biological processes, highlighting the complex interplay between abnormal copper metabolism and immune responses in ALS pathogenesis (111).

Some research indicates that copper metabolism dysfunction in ALS may increase oxidative stress, foster lipid peroxidation and ferroptosis, and thus injure neurons (122). Copper modulates the cell’s antioxidant defenses via interactions with enzymes such as thioredoxin reductase (TRXND) and glutathione peroxidase (GPX) (111). Another study found that copper is associated with membrane lipids such as sphingomyelins and fatty acid acyl glycosides, both essential for neuronal function and integrity (124, 125). Copper imbalance not only heightens oxidative stress but may also interfere with lipid metabolism and signaling, further advancing the neurodegenerative processes in ALS (111).

In one study, tetrathiomolybdate (TTM), used as a copper chelator, lowered spinal-cord copper levels and SOD1 activity in mice, delaying ALS onset and progression and extending survival (126). Another study showed that triazines, also acting as copper chelators, significantly improved survival in an ALS mouse model, suggesting that copper chelation can slow disease progression (127). Ceruloplasmin, a multi-copper oxidase involved in iron metabolism regulation and systemic copper transport, was found to increase in ALS model mice during disease progression, yet its enzymatic activity substantially declined (128). This indicates that ALS-related copper dysregulation not only affects metal loading in a single protein but also systematically reduces the functionality of copper-dependent enzymes, further disrupting metabolic balance in the central nervous system (129).

In the pathogenesis of amyotrophic lateral sclerosis (ALS), copper metabolism imbalance not only affects the function of SOD1 but also plays a key role in regulating immune responses in the central nervous system (CNS), thereby promoting the development of neurodegeneration and the pathological progression of the disease (119).

First, in ALS, the mutant SOD1 interacts with mitochondrial membrane proteins such as Bcl-2 and VDAC1, leading to conformational changes in these proteins that expose their death domains, triggering apoptosis (116). The abnormal activity of VDAC1 also affects the mitochondrial membrane potential and permeability, leading to an increase in intracellular Ca^2+^ concentrations, which further exacerbates the activation of immune cells and the inflammatory response (116).

Secondly, copper imbalance also directly participates in the regulation of immune cell function (119). Studies have shown that copper ions are important regulatory factors for the function of immune cells such as microglia and astrocytes (119). In ALS, excessive copper accumulation promotes the activation of these immune cells, particularly by enhancing their oxidative stress response and the secretion of pro-inflammatory cytokines (130). Copper interacts with antioxidant enzymes such as SOD1 within these immune cells, enhancing the oxidative stress response in neutrophils (123). Cytokines such as interferon-γ (IFN-γ) and tumor necrosis factor-α (TNF-α) secreted by these immune cells not only promote the further expansion of the immune response but also increase copper uptake and accumulation, forming a vicious cycle that exacerbates neuronal damage (111).

Copper metabolism imbalance may also worsen immune responses through mechanisms such as lipid peroxidation (130). In immune responses, oxidative stress is an important pathogenic mechanism (129). The excessive accumulation of copper promotes the generation of reactive oxygen species (ROS) and lipid peroxides, which can directly damage neurons, further activate the immune system, and lead to neurodegeneration (129). Therefore, copper not only alters the function of SOD1, leading to oxidative stress and cell death, but also activates inflammatory responses in immune cells, promoting the progression of ALS (130).

In conclusion, there is a complex interaction between copper metabolism imbalance and the immune response in ALS (111). Copper regulates the function and folding of SOD1, maintaining normal immune cell function, while excessive copper accumulation or deficiency changes immune cell activation and pro-inflammatory responses, leading to increased neuroinflammation, thereby promoting the occurrence of neurodegeneration (116). Copper homeostasis imbalance in ALS affects not only the redox state and immune cell function but also potentially modulates immune responses, influencing the progression of neurodegenerative diseases (123). Restoring copper metabolic balance, especially in the immune response, may provide a new direction for the treatment of ALS and other neurodegenerative diseases (111).

#### Huntington’s disease

Huntington’s disease (HD) is a progressive neurodegenerative condition caused by the expansion of the polyglutamine (polyQ) region in the huntingtin (Htt) protein (131). Symptoms include movement, cognitive, and psychiatric disturbances, typically manifesting in midlife and progressively worsening over the subsequent two decades (132). Current treatments mainly focus on reducing HTT gene expression, nucleic acid therapies, gene therapy, DNA repair, and cell replacement strategies (133).

Research has shown that altering the expression of copper transporters (e.g., Ctr1B and DmATP7) or regulating dietary copper intake can significantly influence HD pathology (131). Both copper excess and copper deficiency affect the aggregation and fibrillization of huntingtin protein (Htt exon1-polyQ), thereby influencing disease progression (131). Studies further indicate that removing potential copper-binding sites on Htt can eliminate copper’s pathogenic effect in HD, suggesting that Huntington’s disease is not only a polyglutamine disease but also involves copper-regulated pathogenic mechanisms (131).

One experiment demonstrated that copper ions markedly enhance the toxic aggregation of mutant huntingtin protein (Htt) associated with HD (134). In a fruit fly model, dietary copper promoted Htt oligomer formation, increased β-amyloid structures, and strengthened the interaction between Ref(2)P and Htt aggregates, contributing to neurotoxicity (134). Copper intensified synaptic degeneration, shown by elevated caspase-3-mediated cell death, reduced BRP expression, and thinner neuronal structures (134). Moreover, increased dietary copper led to more Htt aggregates, especially smaller ones (<5 μm^2^) (135). These results highlight the significant contribution of copper in HD pathogenesis by exacerbating Htt aggregation and neurotoxicity (136). Further research revealed that the copper chelator D-penicillamine (DPA) effectively alleviated this copper-induced Htt aggregation and β-amyloid accumulation, thereby reducing synaptic damage and cytotoxicity (137, 138). These findings provide new insights into copper’s potential role in HD and support copper chelation as a possible therapeutic avenue (134).

Some studies suggest that copper’s interaction with metals like zinc (Zn) may inhibit DMT1, causing imbalances in metals and worsening HD pathology (134, 136). Meanwhile, research indicates that natural antioxidants like rutin can mitigate copper-induced toxicity, reduce protein aggregation, and prevent neurodegenerative injury through chelating effects, suggesting a potential therapeutic application in HD (136).

Studies show that in early-stage HD, especially in the pre-symptomatic phase, copper levels in CSF are elevated while zinc (Zn) levels decrease (139). This rise in copper may reflect changes in blood-brain barrier integrity or metal transport mechanisms (139). Copper directly interacts with mutant huntingtin protein (mHTT), promoting its aggregation and hastening neuronal death (134). Excess free copper ions can increase reactive oxygen species (ROS) levels through the Fenton reaction, enhancing oxidative stress and cellular damage (140). Moreover, copper affects the regulation of neurotransmitters by promoting the synthesis and release of GABA, disturbing inhibitory neurotransmission and worsening HD-related motor and cognitive symptoms (141). Excess copper may also displace zinc in enzymatic sites, altering enzyme activity and interfering with vital biochemical processes such as mitochondrial function and antioxidant defense (139).

Conversely, copper deficiency could reduce its chelation of iron, leading to increased free iron participating in ROS generation and thereby exacerbating oxidative stress (142). Additionally, copper is a key component of cytochrome c oxidase, so inadequate copper might impair mitochondrial function and ATP production, further diminishing energy output (143). Because copper is also an essential cofactor of superoxide dismutase 1 (SOD1), insufficient copper may lower antioxidant activity and render the brain more vulnerable to oxidative damage (144).

An animal study showed that copper supplementation in the drinking water of mice with quinolinic acid (QUIN)-induced HD had a neuroprotective effect (139). After 28 days of copper supplementation, there was no impact on the mice’s food intake, body weight, or water consumption, but striatal copper levels significantly increased (145). Notably, following QUIN-induced injury, copper supplementation elevated striatal copper content compared to the group receiving only QUIN injury, implying a potential protective role for copper in neural damage (146). Copper supplementation prevented QUIN-induced reductions in GABA levels and reduced circling behavior, suggesting copper protects by inhibiting NMDA receptor activity and enhancing antioxidant enzymes (e.g., Cu–Zn SOD) (146). Copper supplementation significantly decreased oxidative stress indicators [e.g., ROS and lipid peroxidation (LP)], supporting the idea that copper mitigates neuronal damage by activating endogenous antioxidant systems (147, 148). Copper showed protective effects in the QUIN-induced HD model, but further research is needed to fully understand its role in HD, particularly whether increased copper levels compensate for free radical damage (146).

The role of copper in Huntington’s disease (HD) goes beyond directly binding to mutant huntingtin (mHTT) and promoting its aggregation; it also involves exacerbating the pathological process through the regulation of immune responses and oxidative stress mechanisms (131). When copper metabolism is disrupted, excessive accumulation of copper generates reactive oxygen species (ROS) via the Fenton reaction (133). These free radicals damage lipids, proteins, and DNA within cells, leading to cell death (133). Additionally, oxidative stress activates immune cells, particularly microglia, which promote neuroinflammatory responses (133). The activation of these immune cells not only promotes further accumulation of copper in the nervous system but also exacerbates local neuronal damage (134).

Specifically, copper accumulation leads to abnormal activation of immune response cells (136). Copper directly affects the function of immune cells, altering their activation, proliferation, and migration behavior (136). When copper levels are excessive, immune cells such as microglia and macrophages become overactivated and release large amounts of pro-inflammatory cytokines, such as interferon-γ (IFN-γ), tumor necrosis factor-α (TNF-α), and interleukins (IL-1, IL-4, etc.) (139). These cytokines not only enhance local inflammation but also further promote copper uptake and accumulation through feedback mechanisms, thereby creating a vicious cycle that exacerbates neuronal damage (134).

Furthermore, copper regulates the expression of metallothioneins (such as MtnA and MtnB), which affect copper homeostasis within cells (131). Metallothioneins are intracellular copper regulators involved in copper absorption, storage, and excretion (131). When copper levels are excessive, the expression of these metallothioneins changes, which may lead to an ineffective cellular response to oxidative stress, further promoting neuronal damage (131). The excessive accumulation of copper not only exacerbates immune responses through these direct oxidative damage pathways but may also influence immune cell function by altering the metal ion balance in immune cells, thus advancing neurodegenerative processes (131).

Copper metabolism disruption may also impact immune responses through interactions with other metals, such as zinc and iron (136). Copper and metals like zinc share the same transport proteins and regulate similar signaling pathways (136). Therefore, excessive copper may compete with zinc for transport channels, altering zinc’s function in immune cells (136). Zinc is an essential metal for various antioxidant enzymes, and its deficiency reduces immune cells’ ability to defend against oxidative stress, thereby worsening immune responses and neuronal damage (136). The interaction between copper metabolism disruption and the imbalance of metals like zinc and iron complicates immune responses, further accelerating the neurodegenerative progression of HD (136).

In summary, copper’s role in HD is not limited to directly promoting mHTT aggregation and toxic expression but also exacerbates neurodegeneration by intensifying oxidative stress, altering immune cell function, and modulating immune responses (131). The excessive accumulation of copper plays a key role in HD’s pathological process through its dual impact on oxidative stress and immune responses (136, 144). Therefore, regulating copper metabolism and immune responses could provide new intervention strategies for HD treatment, especially in terms of copper homeostasis restoration and immune modulation (139).

#### Parkinson’s disease

Parkinson’s disease (PD) is a common neurodegenerative disorder and currently the fastest-growing neurological condition worldwide (149). Its etiology is complex. In 3–5% of cases, single-gene mutations are responsible, while others correlate with genetic risk variants, family history, constipation, nonsmoking status, and exposure to environmental toxins (e.g., pesticides and trichloroethylene) (149). Core manifestations include bradykinesia, resting tremor, and muscular rigidity, but non-motor symptoms (such as constipation, hyposmia, depression, cognitive decline, and sleep disturbances) also significantly impact quality of life (150). PD typically progresses slowly, with non-motor symptoms often appearing years before motor symptoms, leading to delayed diagnosis (151). Diagnosis primarily relies on clinical presentation and is further supported by a positive response to levodopa treatment; additional tests help rule out atypical presentations (152). Current trends in PD diagnosis focus on early detection and individualized approaches (150). Integrative methods—combining highly sensitive technologies, specific molecular markers, and artificial intelligence—aim to improve early diagnostic accuracy and enhance patient treatment and quality of life (153).

The hallmark feature of PD is the gradual degeneration of dopaminergic neurons in the substantia nigra pars compacta (SNpc), leading to reduced dopamine levels in the striatum (particularly in the putamen) and motor symptoms (154). Loss of nigral neurons and the dopaminergic denervation of the basal ganglia underlie the onset of these motor deficits. Decreased expression of the dopamine transporter (DAT), aromatic L-amino acid decarboxylase (AADC), and vesicular monoamine transporter-2 (VMAT-2) in the striatum are important indicators of disease progression (152).

Lewy bodies—intraneuronal inclusions composed primarily of α-synuclein (α-Syn)—are another core pathological hallmark of PD; these α-Syn aggregates (oligomers and Lewy bodies) can be detected in neurons, glial cells, and peripheral tissues (152, 155). The aberrant aggregation of α-Syn leads to neuronal dysfunction and disrupts key processes, including synaptic transmission, lipid metabolism, vesicular transport, and dopamine metabolism, exacerbating neuronal damage (156). Misfolded α-Syn can propagate between neurons, inducing the aggregation of endogenous α-Syn in healthy cells and spreading pathology (157). This mechanism is considered a major driver of PD pathogenesis and interacts with mitochondrial dysfunction, lysosomal pathway abnormalities, and neuroinflammation to facilitate disease progression (158).

To date, six monogenic forms of PD have been identified, arising from mutations in SNCA, LRRK2, Parkin, PINK1, DJ-1, and ATP13A2 (159). Additional genes—such as GBA, MAPT, and APOE—also raise PD risk via polymorphisms (157). The Bax protein promotes dopaminergic neuron apoptosis in PD through multiple mechanisms, including interactions with antiapoptotic Bcl-2 family proteins and translocation to the mitochondrial membrane to inhibit other antiapoptotic factors; this mechanism may function independently of cytochrome c release (160). Bax can exert either proapoptotic or antiapoptotic effects in different cell types, acting as a critical cofactor in the apoptotic cascade (161).

A randomized controlled trial demonstrated that copper levels in dopaminergic neurons of the substantia nigra are marginally lower in PD patients than in controls (162). Another mouse study showed that copper levels are abnormally elevated during the prodromal stage of PD and that ATH434 can regulate copper homeostasis and improve olfactory function (163). As the disease progresses, metal dyshomeostasis in PD may shift from copper-dominated abnormalities to iron-centered pathology (163). Copper-chelating agents like PBT2 and TDMQ20 bind and transport excessive copper in the brain, reducing copper toxicity while preserving essential levels for normal neuronal physiology (164). These chelators can partially reverse or slow pathological damage associated with Aβ aggregation and PD (165). Proteins such as FDX1, DLAT, and LIAS play pivotal roles in the tricarboxylic acid (TCA) cycle; these proteins can undergo lipoylation and, under conditions of copper excess, abnormally aggregate, triggering cell death (166).

Research suggests that ceruloplasmin (Cp) is centrally involved not only in copper and iron metabolism in the liver and bloodstream but also in regulating metal homeostasis and neuronal function in the brain (167). Cp deficiency and mutations in copper-transporting genes such as ATP7B can cause abnormal metal distribution in the brain and induce (or exacerbate) neurodegenerative processes, including PD (168). Genes such as ATP7A, SLC31A1, and DBT, closely linked to copper homeostasis, neuronal function, and immunometabolism, display significantly altered expression in the substantia nigra of PD patients, correlating with disease staging (169). Other studies indicate that ATP7B maintains cellular copper homeostasis by transporting copper to the Golgi apparatus for integration into copper-dependent enzymes, NFE2L2 (NRF2) activates various antioxidant enzymes to mitigate oxidative stress and inflammation caused by copper excess, and MTF1 detects abnormal metal concentrations and induces detoxification and stress-response gene expression (170). These three factors together form a core network for copper regulation; dysfunctional expression or activity can exacerbate PD and other neurodegenerative diseases (170, 171).

α-Synuclein (α-Syn) binds Cu^2+^ with high affinity via its N-terminal amine, the side chain of Asp2, and the imidazole group of His50, inducing conformational changes that modulate copper redox activity, reduce oxidative stress, and promote membrane recycling and SNARE-complex membrane fusion (172). In PD, Cu^2+^ binding may weaken the interaction of α-Syn with membranes, increasing its soluble fraction, accelerating fibrillization, and promoting Lewy body formation, thereby driving disease progression (172). Copper ions have a high affinity for α-Syn, enhancing α-Syn fibril formation and directly leading to cytotoxicity or inducing α-Syn aggregation (173). Studies show that copper ions can promote the formation of α-Syn short fibrils (<0.2 μm) via an atypical pathway; these short fibrils regulated by copper display heightened intracellular transmissibility and toxicity (174). Other research has found that negatively charged membrane proteins, such as heparan sulfate proteoglycans (HSPGs), mediate copper/iron-induced fibril internalization via electrostatic interactions (175). Concurrently, changes in α-Syn secondary structure and fibril morphology induced by copper/iron ions enhance its aggregation and cellular toxicity (175). Clinically used chelators TETA and DF effectively hinder the damaging effects of copper/iron ions on α-Syn fibril propagation and prolong the lifespan of PD model nematodes (175). Furthermore, copper/iron ions have been found to affect the aggregation or toxicity of other disease-associated amyloid proteins (including β-amyloid, tau, and prion proteins), suggesting new avenues for investigating other protein misfolding disorders (175).

Copper ions (Cu^2+^) alter α-Syn conformation and aggregation by directly binding to critical residues (His50 and Asp121) and promoting reactive oxygen species (ROS) generation (176, 177). Studies indicate that upon Cu^2+^ binding, the hydrophobic non-amyloid-β component (NAC) domain becomes more solvent-accessible, facilitating initial hydrophobic interactions that form toxic oligomers (178, 179). Moreover, Cu^2+^ binding can disrupt long-range intramolecular interactions (e.g., His50-Val71 and Asp121-Lys96), weakening the barrier effect among NAC domains and accelerating α-Syn fibrillization (180). Copper binding results in a more compact α-Syn peptide chain, reinforcing intramolecular hydrogen bonds and residue interactions (181). Such changes likely promote protein aggregation in PD by influencing the tightness of the NAC (non-amyloid-β component) region and the affinity of the peptide chain for membranes (182).

Studies demonstrate that copper triggers abnormal aggregation by directly binding lipoylated proteins, leading to protein toxicity stress and cell death (183). During this process, lipoylated TCA-cycle enzymes are key targets that, under copper’s influence, aggregate and destabilize iron–sulfur (Fe–S) cluster proteins, ultimately compromising mitochondrial metabolic function (184, 185). Glutathione (GSH), a natural intracellular copper chaperone, is critical in this context (186). When GSH is depleted, cells become markedly more sensitive to copper, presenting decreased lipoylation, increased DLAT aggregation, and further destabilization of Fe–S cluster proteins (8). These changes induce severe protein toxicity stress and cell death (8). An animal study using a novel fluorescent probe (R13) showed significantly reduced GSH levels in the brains of a PD mouse model, whereas oxidized glutathione (GSSG) levels increased, indicating that PD is associated with oxidative stress and that lower GSH levels reflect weakened radical-scavenging capacity (187). Other research has demonstrated that polymorphisms in GST (glutathione S-transferase) are closely related to PD risk; specifically, the deletion (null) genotypes of GSTM1 and GSTT1 significantly increase PD risk, and a combined deletion further heightens this association (188). In animal experiments, copper ions binding to GSH caused intracellular GSH depletion, impairing antioxidant capacity, exacerbating oxidative stress and protein nitration, and leading to neuronal damage (8). Meanwhile, GSH depletion disrupts the ubiquitin-proteasome system (UPS) and autophagic pathways, resulting in aggregation of proteins such as α-Syn—an important mechanism of neuronal injury in PD (189).

DLAT is a key enzyme in protein lipoylation and plays a vital role in the TCA cycle (8). Under copper overload, DLAT expression significantly increases, promoting abnormal protein lipoylation and causing aggregated lipoylated proteins as well as the loss of iron–sulfur (Fe–S) proteins (190). These changes induce protein toxicity stress, further exacerbating cell apoptosis and tissue damage (148). Studies show that 4-amino-TEMPO (4-AT) provides antioxidant effects and may activate Nrf2, enhancing cellular antioxidant capacity and restoring GSH levels to improve neuronal viability (191). Another study found that hypoxanthine boosts cysteine uptake via EAAC1 (a cysteine transporter) in HEK293 and SH-SY5Y cells, increasing intracellular GSH synthesis and exhibiting neuroprotective effects against oxidative stress (e.g., H₂O₂ treatment) (192).

The relationship between copper metabolism abnormalities and central nervous system (CNS) immune responses has gained increasing attention in the pathological mechanisms of Parkinson’s disease (PD) (151). One of the pathological features of PD is the loss of dopaminergic neurons and the accumulation of Lewy bodies, with neuroinflammation being considered a key factor in this process (149). Abnormal copper accumulation plays an important role in PD pathology, and through its regulation of the immune system, particularly its impact on immune cells, it enhances neuroinflammatory responses and further exacerbates neurodegenerative damage (154).

Firstly, abnormalities in copper metabolism lead to the overactivation of immune cells, especially microglia and peripheral immune cells such as macrophages, monocytes, and neutrophils (168). Studies have shown that copper accumulation can stimulate microglial activation, as these cells are the primary immune cells in the CNS and play a critical role in neuroinflammation (168). Overactivation of microglia releases inflammatory cytokines, further promoting neuronal damage and exacerbating neurodegenerative lesions (170). The infiltration and activation of peripheral immune cells are also regulated by copper, which increases their ability to enter the CNS, thus intensifying the local inflammatory response (170). Copper not only directly affects the functions of these immune cells but also regulates their migration and activation, further driving the progression of PD (182).

Using the 6-hydroxydopamine (6-OHDA) mouse model, combined with TSPO PET and TREM1 PET imaging techniques, researchers further explored the role of copper in PD. TSPO is a marker for microglia and peripheral immune cells (193). The study found that copper accumulation is closely related to the activation of immune cells in the 6-OHDA mouse model (193). TREM1 PET imaging showed that the infiltration of peripheral immune cells, particularly neutrophils, enhanced this process, suggesting that the role of copper in immune activation should not be underestimated (193). The high expression of TREM1 as a pro-inflammatory immune cell marker, correlated with copper metabolism abnormalities, further reveals that copper may enhance neuroinflammatory responses by influencing immune cell dynamics, thus promoting the neurodegenerative process of PD (193).

Additionally, copper imbalance also affects the metal homeostasis within neurons, influencing the aggregation of α-synuclein (152). Studies have shown that copper can bind to α-synuclein and promote its aggregation, forming Lewy bodies, which is a hallmark pathological feature of PD (156). Copper accumulation not only promotes α-synuclein aggregation but also induces oxidative stress, further enhancing neuroimmune responses (157). The aggregation of α-synuclein promotes neuroinflammation, and this immune activation is closely related to copper regulation (170).

In conclusion, copper metabolism imbalance not only directly affects the functions of immune cells but also influences the aggregation of α-synuclein by altering metal homeostasis within neurons, thereby exacerbating neuroinflammation and neurodegenerative lesions (172). Copper accumulation drives neuronal damage and PD progression through immune cell activation and increased oxidative stress (175). This finding reveals the complex interplay between copper and immune responses, providing potential therapeutic targets for future PD treatment strategies, particularly in copper homeostasis regulation and immune response control (182).

#### Stroke

Stroke is caused by acute focal injury to the central nervous system (CNS), leading to neurological deficits from vascular lesions (194). Strokes are generally classified as ischemic or hemorrhagic; the vast majority are ischemic, attributable to reduced blood flow (usually due to arterial occlusion) (195). During stroke, blood flow is interrupted or a cerebral artery ruptures, discontinuing energy supply to brain tissue (194). This interruption results in metabolic imbalance within neurons and heightened oxidative stress responses (194). In this process, copper contributes to neuronal damage and death via multiple mechanisms, closely tied to reactive oxygen species (ROS) production, mitochondrial dysfunction, and regulation of apoptotic signaling pathways (196).

Copper serves as an essential cofactor for numerous redox enzymes—including superoxide dismutase (SOD)—that are critical for redox balance within neurons (197). When copper levels are dysregulated (deficient or excessive), ROS levels surge, collapsing mitochondrial membrane potential and accumulating ROS (198). Excessive ROS generation triggers lipid peroxidation, protein damage, and DNA breaks, initiating endogenous apoptotic pathways (2). These effects are particularly pronounced in ischemic stroke: oxygen and nutrient supply plummet, forcing neurons to rely on anaerobic glycolysis for ATP production, an inefficient pathway insufficient to meet metabolic demands (199). This shortfall further worsens intracellular calcium overload and redox imbalance (200).

Studies show that SOD1 overexpression substantially reduces ROS levels by enhancing the antioxidant capacity of neural stem cells (NSCs) against ischemia-reperfusion injury (201). Consequently, SOD1 overexpression lessens oxidative stress-mediated cell death and improves NSC survival and reparative capacity for ischemic brain damage both *in vitro* and *in vivo* (201). In addition to promoting vascular endothelial growth factor (VEGF) secretion and angiogenesis in ischemic regions, SOD1 overexpression supports the release of neuroprotective factors, reinforcing endogenous repair mechanisms that shrink infarct volume and improve brain function (202).

Research suggests that combined deficiency of copper-transporting proteins (e.g., ATP7A) and metallothioneins (MTs) abolishes cellular tolerance to copper toxicity (203, 204). Even at extremely low copper concentrations, these cells experience severe copper overload, resulting in rapid ROS accumulation, collapse of the mitochondrial membrane potential, and disruption of the glutathione (GSH) redox system, ultimately activating various oxidative stress-mediated cell death pathways (e.g., apoptosis, necrosis, or cuproptosis) (205). Another study indicates that high mobility group protein B1 (HMGB1), acting as an inflammatory mediator induced by cuproptosis, is rapidly released extracellularly post-cerebral ischemia (206). HMGB1 then activates danger-associated molecular pattern (DAMP) signaling, triggering microglial activation and proinflammatory cytokine release, thereby intensifying neuroinflammation and causing further disruption of the blood-brain barrier (205).

Metallothioneins (MTs) and the glutathione (GSH) system both modulate copper toxicity through their antioxidant properties following cerebral ischemia (207). MTs bind free copper via thiol groups, preventing copper ions from damaging other intracellular molecules, whereas GSH maintains redox homeostasis by binding copper ions (27). Under ischemic conditions, GSH is rapidly oxidized, reducing the GSH/GSSG ratio and weakening antioxidant capacity, rendering cells more vulnerable to the combined damage of copper and ROS (205). Additionally, copper overload inhibits the synthesis of iron–sulfur cluster proteins, further amplifying metabolic imbalance (208).

In ischemic stroke, autophagy can serve as a protective mechanism, clearing damaged mitochondria and proteins; yet excessive autophagic activation may hasten cell death (209). Copper modulates autophagy levels through the AMPK-mTOR axis and also directly binds to and activates autophagy-related kinases ULK1/ULK2. In cerebral ischemia, copper-driven autophagy may not only counter cellular damage but also exacerbate neuronal loss by selectively degrading antiapoptotic or antioxidant factors such as GPX4 (205).

Copper regulation of inflammatory responses is closely interwoven with stroke pathogenesis (210). Studies suggest that copper-mediated redox reactions may escalate inflammatory responses by promoting proinflammatory cytokine release (210, 211). In the setting of ischemia-reperfusion injury, copper accumulation may activate the NF-κB signaling pathway, intensifying inflammatory cascades (212). Moreover, disrupted copper metabolism can undermine extracellular matrix remodeling around blood vessels, triggering cerebrovascular dysfunction (213). For instance, copper enhances lysyl oxidase (LOX) activity, promoting collagen cross-linking and increasing vascular stiffness (214). This alteration compromises the integrity of the blood-brain barrier, creating a potential pathological foundation for vascular stroke (2).

Copper has dual roles in modulating excitotoxicity and neuronal death (215). By regulating N-methyl-D-aspartate receptor (NMDAR) activity, copper influences glutamate neurotoxicity (216). Under normal physiological conditions, copper released at the synapse suppresses NMDAR activity, thus exerting neuroprotective effects (216). However, in the presence of copper deficiency or ATP7A dysfunction, this regulatory mechanism falters, leading to NMDAR overactivation, calcium overload, and neuronal apoptosis (217, 218). In hemorrhagic stroke or following brain trauma, copper can accumulate directly in brain tissue, generating harmful hydroxyl radicals through the non-enzymatic Fenton reaction, which induces apoptosis or necrosis (219). Furthermore, copper can bind directly to key proteins (e.g., prion protein, Aβ, or tau), forming abnormal aggregates that magnify brain tissue injury in a vicious cycle (2).

Copper’s functions are not limited to the onset of brain injury but also extend to neurological recovery (220). Copper participates in the maturation of various neuropeptides, including neuropeptide Y and corticotropin-releasing hormone, both of which are vital for neuroprotection and modulating neuroinflammation after stroke (196, 199). Additionally, copper-dependent metalloproteins play regulatory roles in angiogenesis and neural regeneration, influencing the functional remodeling of brain tissue (2).

Clinically, modulating copper metabolism abnormalities shows therapeutic promise (221). Under copper deficiency, copper supplementation restores copper-dependent enzyme function and bolsters antioxidant defenses (196, 222). Conversely, when copper is elevated or abnormally localized, copper-chelating agents (e.g., penicillamine or trientine) alleviate oxidative stress and neurotoxicity by reducing copper load (2). The fine-tuned regulation of copper metabolism and distribution in the brain, via lipophilic metal-protein-attenuating compounds (MPACs) that redistribute copper, might be a potential strategy for treating stroke and related neurodegenerative diseases (71, 223). This approach provides an avenue to intervene in copper metabolism and precisely modulate copper distribution to restore brain function and reduce pathological damage (2). Thus, targeted copper regulation provides new avenues for clinical therapy (71).

An animal study showed that in a mouse model treated with tetrathiomolybdate (TM), microvessel density (MVD) decreased by 50% (224). A case-control study indicated a nonlinear, L-shaped relationship between increased copper intake and reduced stroke risk: stroke risk gradually fell with greater copper intake before leveling off beyond a certain intake threshold (225). Other research has found that elevated plasma copper concentration is significantly associated with increased ischemic stroke risk (226). The same study also discovered a strong correlation between plasma copper levels and hyperlipidemia, a known risk factor for carotid atherosclerosis and ischemic stroke (227). A meta-analysis corroborated these findings: across eight high-quality studies, serum copper levels were significantly higher in patients with ischemic stroke than in controls (228).

An animal experiment showed that mice consuming trace amounts of copper (one-tenth the EPA’s upper limit) for 14 weeks had increased infarct volume and worse neurologic outcomes in a MCAO-induced ischemic brain injury model (229). This damage was closely tied to endothelial progenitor cell (EPC) dysfunction, as copper intake impaired EPC migration, tube formation, and adhesion, while markedly reducing phosphorylated eNOS and MnSOD levels, lowering nitric oxide (NO) production, and raising TSP-1 expression—ultimately inhibiting angiogenesis (230–232). Brain tissue analyses revealed that copper-exposure-induced EPC dysfunction led to significantly lower microvessel density in ischemic regions, hindering vascular growth factor (e.g., VEGF) secretion and endothelial repair mechanisms, and aggravating ischemic brain injury (229, 233, 234). These findings illuminate the core molecular and cellular processes by which trace copper intake exacerbates cerebral ischemic damage (229).

Copper plays a key role in immune responses in the central nervous system (CNS), primarily through its involvement in redox reactions and its regulation of immune system function (196). Copper not only participates in neurotransmitter synthesis, energy metabolism, and antioxidant defense but also directly influences immune responses (198). When copper metabolism is disrupted, either through excess copper or copper deficiency, it can trigger abnormal immune responses, thereby exacerbating neuronal damage, especially in neurodegenerative diseases such as ischemic stroke (200).

Firstly, the role of copper in the immune response within the nervous system is closely related to oxidative stress (2). Copper, through its participation in redox reactions, helps maintain the redox balance within cells (2). For example, copper acts as a cofactor for antioxidant enzymes such as copper/zinc superoxide dismutase (Cu/Zn SOD), helping to clear reactive oxygen species (ROS) and thus mitigating oxidative damage (2). However, excessive copper accumulation leads to increased ROS generation, resulting in oxidative stress and cellular damage (196). This oxidative stress not only directly harms neurons but also activates immune cells in the CNS, particularly microglia, which play a critical role in neuroinflammation (227). Excess copper accumulation can stimulate microglial activation, prompting the release of more pro-inflammatory cytokines such as TNF-α and IL-1β, which further exacerbate local neuroinflammatory responses (235).

The mechanism by which copper excess activates immune cells through oxidative stress is especially important during stroke (196). Oxidative stress increases the accumulation of free radicals and activates signaling pathways such as MAPK and NF-κB, promoting the production of pro-inflammatory cytokines (196). In this process, copper exacerbates oxidative damage by enhancing ROS generation and promoting the overactivation of immune cells, further amplifying local inflammation (2). These interactions create a vicious cycle, where the continuously enhanced inflammatory response not only causes neuronal damage but also accelerates the development of neurodegenerative lesions (196).

In addition to oxidative stress, copper’s role in immune modulation within the nervous system is also crucial (2). During neuro-pathological conditions like stroke, copper homeostasis directly impacts immune cell function (205). Copper regulates the intensity of immune responses through interactions with immune-modulatory factors such as metallothioneins (MTs) (2). When copper is deficient, the activation of microglia and immune responses is suppressed, leading to a weakened immune response and making the nervous system more vulnerable to damage (196). In contrast, excess copper enhances immune cell activation and pro-inflammatory cytokine release, thus promoting neuroinflammation and accelerating pathological progression (196).

Furthermore, the neuroimmune mechanisms related to stroke also involve copper’s relationship with atherosclerosis (227). Excess copper accumulation may promote lipid deposition in blood vessel walls through the oxidation of low-density lipoprotein (LDL), leading to the development of atherosclerosis (229). Copper exacerbates vascular damage through this mechanism and may also stimulate immune responses in the vascular endothelium, increasing local inflammation and thereby raising the risk of ischemic stroke (228).

Dysregulation of copper metabolism, especially copper excess, has been confirmed as a significant factor in the occurrence and progression of neurodegenerative diseases like stroke (235). Excess copper not only causes direct oxidative damage but also alters immune cell function, changing the neuroimmune response and further promoting inflammation within the nervous system (235). Moreover, maintaining copper homeostasis is crucial for normal immune function in the CNS, protecting neurons from damage, and promoting cerebrovascular repair (228). Disrupted copper metabolism may become a potential factor in the worsening of neurodegenerative diseases and cerebral ischemic injuries, suggesting that special attention should be given to copper balance in the treatment of stroke and other neuroimmune diseases (227). Proper copper intake and homeostasis are vital for stroke prevention, neuronal damage repair, and the normal regulation of immune responses (229).

#### Condensing content

Copper homeostasis is crucial for normal physiological functioning, playing pivotal roles in energy metabolism, antioxidant defense, and immune regulation, while also being intimately involved in the onset and progression of neurodegenerative diseases. The recent conceptualization of cuproptosis has elevated copper from its conventional classification as an “oxidative stress factor” and “enzyme cofactor” to a central determinant of cell fate. Existing studies indicate that in the central nervous system, copper can drive protein misfolding and aggregation to exacerbate neuronal injury, but it can also participate in the activation of antioxidant enzymes and repair factors to inhibit excessive cell death and inflammation, demonstrating a “double-edged sword” effect. As research into the pathological mechanisms of Alzheimer’s disease, Parkinson’s disease, amyotrophic lateral sclerosis, Huntington’s disease, and stroke intensifies, emerging causal connections between copper homeostasis and neuronal damage, protein aggregation, mitochondrial dysfunction, and immune-inflammatory processes provide new insights into critical disease pathogenesis and clinical intervention strategies (72, 119, 131, 157, 196). Nevertheless, the precise mechanisms and pathological significance of cuproptosis in neurodegenerative conditions require further investigation. First, copper dysregulation involves dynamic, multilayered regulation—encompassing transmembrane transport, chaperone-mediated protein allocation, and redox coupling—leaving many key regulatory factors and interactions incompletely understood. Second, cuproptosis may intersect with ferroptosis, apoptosis, and autophagy, potentially synergizing to amplify damage, underscoring the importance of clarifying its predominant role at various disease stages for targeted therapy and prognostic assessment. Third, current evaluations of interventions such as copper chelators, metal-protein-attenuating compounds (MPACs), and gene therapy remain constrained by dosage windows, blood-brain barrier permeability, and individual variability, necessitating large-scale clinical trials to confirm their safety and efficacy. Future research might focus on real-time, high-resolution molecular imaging and multi-omics platforms that monitor copper dynamics in the brain and its binding states with critical proteins, on systematically screening and optimizing cellular and animal models to identify drug targets and small-molecule compounds that can balance removal of excess copper or replenishment of copper deficiency without disrupting the overall metal equilibrium, and on leveraging gene editing and immune modulation to reconstruct copper homeostasis and inflammatory pathways in the neural microenvironment for personalized therapies. As our understanding of cuproptosis and the pathophysiology of the nervous system deepens, interventions centered on copper homeostasis may pave the way for next-generation treatments of neurodegenerative diseases, offering novel possibilities for slowing disease progression and improving patient quality of life.
