# Correction: From copper homeostasis to cuproptosis: a new perspective on CNS immune regulation and neurodegenerative diseases

**DOI:** 10.3389/fneur.2025.1664184

**Published:** 2025-07-29

**Authors:** Luhao Li, Liangzhen Lv, Zhaodi Wang, Xianbao Liu, Qingyi Wang, Hui Zhu, Bei Jiang, Yapeng Han, Xue Pan, Xueming Zhou, Li Ren, Zhuo Chang

**Affiliations:** ^1^Second Clinical Medical School, Heilongjiang University of Chinese Medicine, Harbin, China; ^2^Graduate School, Heilongjiang University of Chinese Medicine, Harbin, China; ^3^Yichun Central Hospital, Yichun, Heilongjiang, China; ^4^Yueyang Hospital of Integrated Traditional Chinese and Western Medicine, Shanghai University of Traditional Chinese Medicine, Shanghai, China; ^5^School of Basic Medical Sciences, Heilongjiang University of Chinese Medicine, Harbin, China; ^6^Third Affiliated Hospital, Beijing University of Chinese Medicine, Beijing, China; ^7^Shunde Women and Children's Hospital, Guangdong Medical University, Foshan, China

**Keywords:** copper homeostasis, cuproptosis, neurodegenerative diseases, immune response, cell death mechanisms

In the published article, author Xianbao Liu was erroneously assigned to affiliation “Graduate School, Heilongjiang University of Chinese Medicine, Harbin, China.” The correct affiliation for author Xianbao Liu is “Yichun Central Hospital, Yichun, Heilongjiang, China.”

The original version of this article has been updated.

